# Systematic comparison of resilience scales using retrospective reports: A practical case study using South African data

**DOI:** 10.4102/ajopa.v6i0.150

**Published:** 2024-07-17

**Authors:** Charles H. van Wijk

**Affiliations:** 1 Department of Industrial Psychology (Mil), Faculty of Military Science, Stellenbosch University, Saldanha, South Africa; 2 Institute for Maritime Medicine, Simon’s Town, South Africa

**Keywords:** COSMIN guidelines, dispositional resilience, hardiness, mental toughness, systematic comparison, validity

## Abstract

The availability of different scales measuring similar constructs challenges scientists and practitioners when it comes to choosing the most appropriate instrument to use. As a result, systematic comparison frameworks have been developed to guide such decisions. The Consensus-based Standard for the Selection of Health Measurement Instruments (COSMIN) is one example of such a framework to examine the quality of psychometric studies. This article aimed, firstly, to explore the psychometric characteristics of resilience measures used in the South African Navy (SAN), in that context. Secondly, it aimed to illustrate the application of the COSMIN guide for comparing psychometric scales and employing data from the aforementioned resilience measures, as a practical case study. The study drew on both published and unpublished data from seven SAN samples, using eight psychometric scales associated with resilience. It assessed structural validity, construct validity, internal reliability and predictive ability. The outcomes were tabulated, and the COSMIN criteria were applied to each data point. All eight scales provided some degree of evidence of validity. However, it was at times difficult to differentiate between the scales when using the COSMIN guidelines. In such cases, more nuanced criteria were necessary to demonstrate more clearly the differences between the psychometric characteristics of the scales and ease in subsequent decision-making.

**Contribution:**

This article illustrated the application of COSMIN guidelines to systematically compare the quality of psychometric study outcomes on local South African data. It further offered evidence of validity for a range of resilience-related measures in a South African context.

## Introduction

Military personnel – whether soldiers or sailors – are exposed to a range of potentially adverse experiences during both training and operational deployments, with a strong requirement to ‘carry on’, or persevere, in spite of hardships and discomfort. Similar demands may also apply to emergency workers (medical staff, fire-and-rescue services, etc.) and police service personnel. This has resulted in calls for local military psychologists to focus not only on psychopathology and its antecedents, such as understanding what went wrong in people’s adaptation to their experiences, but also on their strengths. For instance, they should explore how military personnel adapt, and even thrive when faced with adversity (Bester, 2022; Matthews, 2008; Van Wijk & Waters, 2003).

Many psychological constructs – including resilience – can be assessed by multiple psychometric instruments. This poses a challenge when it comes to choosing the most appropriate instrument for a particular construct of interest. Systematic comparison frameworks can assist in making this decision. One example is the Consensus-based Standard for the Selection of Health Measurement Instruments (COSMIN) risk-of-bias checklist (Mokkink et al., 2018; Prinsen et al., 2018), which examines the quality of psychometric studies. This article offers a practical case study, employing both published and unpublished data on resilience measures used in the South African Navy (SAN), to illustrate the process of comparing psychometric scales.

### Psychological resilience

Psychological resilience is defined as the process of adapting well to adversity, trauma, tragedy, threats or significant sources of stress (American Psychological Association [APA], 2023a). It refers to those qualities that enable a person to withstand adversity, bounce back after setbacks, and adapt successfully to change (Connor & Davidson, 2003).

Resilience is closely associated with (1) biological markers and genetic profiles (Charney, 2004), (2) innate disposition, (3) access to resources, including both financial and social support (APA, 2023b) and (4) developed skills, learned through life experience and specific skills training. The respective contributions of these factors to successful adaptation during life have not yet been fully clarified; this article focusses specifically on dispositional resilience.

Dispositional resilience refers to those intrinsic characteristics that allow people to overcome hardships and even thrive in the face of these (Richardson, 2002; Sagone & De Caroli, 2014). This internal trait allows individuals to work constructively though life’s adversities and is further considered a predictor of both adaptation to stress or trauma, and subsequent mental health (Luthar & Brown, 2007; Maddi, 2002). It has been operationalised in constructs such as a sense of coherence, hardiness, and mental toughness, all located in the domain of positive psychology (Antonovsky, 1987; Clough et al., 2002; Kobasa, 1979). Such constructs of resilience are often considered dispositional, as they represent consistent approaches to life that develop over time. Dispositional resilience is thus sometimes equated to terms such as ‘life orientation’ or ‘worldview’.

Hardiness is a psychological orientation associated with people who remain healthy and continue to perform well in a range of stressful conditions (Arendse et al., 2020; Bartone et al., 2008; Kobasa et al., 1982). Hardiness is considered a construct with three facets, namely commitment, control and challenge (Kobasa, 1979), and hardy individuals appear more resistant to the adverse effects of personal and environmental stress than less hardy individuals (Bartone et al., 2008; Kobasa et al., 1982).

Mental toughness is another term that entails positive psychological resources (Lin et al., 2017). It is a psychological orientation associated with perseverance, mental health and coping strategies (Gerber et al., 2013, 2015; Giles et al., 2018; Gucciardi et al., 2016; Kaiseler et al., 2009; Lin et al., 2017; Mutz et al., 2017). A number of mental toughness models have been developed. For example, the model of Clough et al. (2002) is partially derived from the theoretical foundations of hardiness, with a fourth facet included, namely confidence, whereas Gucciardi et al. (2015) drew on theories of stress and personal resources to develop a unitary model of mental toughness.

### Resilience in military settings

Resilience, and its related dispositional constructs, have been of particular interest in military contexts, given the challenges of military service and associated environmental exposures. Among others, the ability to be resistant to the effects of context-specific stress, as well as the ability to persevere in spite of adversity, appear supportive of adjustment and mental health.

Resilience and related constructs, in particular hardiness, have been shown to influence psychological outcomes among soldiers in training, combat duty and peacekeeping, across various national contexts (Bartone, 1996, 1999; Bartone et al., 2002; Johnsen et al., 2013). There is evidence that hardier soldiers are less likely to develop post-traumatic stress disorder and other mental health conditions after exposure to combat and that they may adapt better both during and after operational deployments (Bartone, 1999, 2000; Britt et al., 2001; Escolas et al., 2013; Pietrzak et al., 2009). Mental toughness has also been associated with performance in military contexts (Godlewski & Kline, 2012; Gucciardi et al., 2015, 2021; Lin et al., 2017). A recent meta-analysis identified a wide range of resilience measures regularly used in military contexts, with the Dispositional Resilience Scale (DRS) arguably the most popular (Van Der Meulen et al., 2020).

### Framework for systematic comparisons of resilience measures

The magnitude of available measures to quantify resilience-related constructs makes it challenging to choose the most appropriate tool for a particular context. Scales can be compared by means of prospective comparative studies, but these are associated with obstacles such as cost, access and so forth. Retrospective data are often more readily available and can be evaluated using systematic comparison frameworks.

The COSMIN checklist (Mokkink et al., 2018) examines the quality of psychometric studies across 10 sections (scale development, content validity, structural validity, internal consistency, cross-cultural validity, reliability, measurement error, criterion validity, hypothesis testing for construct validity, and responsiveness). The COSMIN guidelines further provide parameters for the quality appraisal of reported measurement properties (Farnsworth et al., 2022; Prinsen et al., 2018). An abbreviated description of the COSMIN guidelines has been provided in Table 1. Potential ratings include sufficient (+), insufficient (–) or indeterminant (?) based on the strength of the reported measurement property (Farnsworth et al., 2022).

**TABLE 1:** Updated criteria for good measurement properties.

Measurement property	Rating	COSMIN criteria guideline	More stringent criteria
Structural validity	+	CTT	
		CFA: CFI or TLI or comparable measure > 0.95 OR RMSEA < 0.06 OR SRMR < 0.08^a^	χ^2^/*df* < 3 (‡)^f^ < 5 (±) ≥ 5 (x)
		IRT/Rasch	
		No violation of unidimensionalityb: CFI or TLI or comparable measure > 0.95 OR RMSEA < 0.06 OR SRMR < 0.08	
		*AND*	RMSEA < 0.6 (‡) < 0.8 (±) ≥ 0.8 (x)
		No violation of local independence: residual correlations among the items after controlling for the dominant factor < 0.20 OR Q3’s < 0.37	
		*AND*	
		No violation of monotonicity: adequate looking graphs OR item scalability > 0.30	CFI ≥ 0.95 (‡) ≥ 0.90 (±) < 0.90 (x)
		*AND*	
		Adequate model fit:	
		IRT: χ^2^ > 0.001	
		Rasch: infit and outfit mean squares ≥ 0.5 and ≤ 1.5 OR Z-standardised values > −2 and < 2	
	?	CTT: not all information for ‘+’ reported IRT/Rasch: model fit not reported	
	−	Criteria for ‘+’ not met	
Internal consistency	+	At least low evidence^c^ for sufficient structural validity^d^ AND Cronbach’s alpha(s) ≥ 0.70 for each unidimensional scale or subscale	Cronbach’s α / McDonald’s ω ≥ 0.85 (‡) ≥ 0.80 (±) < 0.80 (x)
	?	Criteria for ‘At least low evidence^c^ for sufficient structural validity^d^’ not met	
	−	At least low evidence^c^ for sufficient structural validity^d^ AND Cronbach’s alpha(s) < 0.70 for each unidimensional scale or subscale	
Reliability	+	ICC or weighted Kappa ≥ 0.70	
	?	ICC or weighted Kappa not reported	
	−	ICC or weighted Kappa < 0.70	
Measurement error	+	SDC or LoA < MIC^d^	
	?	MIC not defined	
	−	SDC or LoA > MIC^d^	
Hypothesis testing for construct validity	+	The result is in accordance with the hypothesis^e^	Correlation with other resilience scales *r* ≥ 0.50 (‡) *r* ≥ 0.35 (±) *r* < 0.35 (x)
	?	No hypothesis defined (by the review team)	Correlation with scales of common mental disorders *r* ≥ 0.40 (‡) *r* ≥ 0.25 (±) *r* < 0.25 (x)
	−	The result is not in accordance with the hypothesis^e^	Differentiate between participants with/out clinical diagnoses Cohens *d* ≥ 1.0 (‡) *d* > 0.8 (±) *d* ≤ 0.8 (x)
Cross-cultural validity/measurement invariance	+	No important differences found between group factors (such as age, gender and language) in multiple group factor analysis OR no important DIF for group factors (McFadden’s *R*^2^ < 0.02)	
	?	No multiple group factor analysis OR DIF analysis performed	
	−	Important differences between group factors OR DIF was found	
Criterion validity	+	Correlation with gold standard ≥ 0.70 OR AUC ≥ 0.70	
	?	Not all information for ‘+’ reported	
	−	Correlation with gold standard < 0.70 OR AUC < 0.70	
Responsiveness	+	The result is in accordance with the hypothesis^f^ or AUC ≥ 0.70	AUC ≥ 0.85 (‡) ≥ 0.80 (±) < 0.80 (x)
	?	No hypothesis defined (by the review team)	OR ≥ 1.5 (‡) ≥ 1.2 (±) < 1.2 (x)
	−	The result is not in accordance with the hypothesis^f^ or AUC < 0.70	Beta > 0.30 (‡) > 0.20 (±) ≤ 0.20 (x)

*Source:* Adapted from Prinsen, C.A., Mokkink, L.B., Bouter, L.M., Alonso, J., Patrick, D.L., De Vet, H.C., & Terwee, C.B. (2018). COSMIN guideline for systematic reviews of patient-reported outcome measures. *Quality of Life Research, 27*, 1147–1157. https://doi.org/10.1007/s11136-018-1798-3; Prinsen, C.A., Vohra, S., Rose, M.R., Boers, M., Tugwell, P., Clarke, M., Williamson, P.R., & Terwee, C.B. (2016). How to select outcome measurement instruments for outcomes included in a ‘core outcome set’ – A practical guideline. *Trials, 17*(1), 449. https://doi.org/10.1186/s13063-016-1555-2; Terwee, C.B., Bot, S.D., De Boer, M.R., Van Der Windt, D.A., Knol, D.L., Dekker, J., Bouter, L.M., & De Vet, H.C.W. (2007). Quality criteria were proposed for measurement properties of health status questionnaires. *Journal of Clinical Epidemiology, 60*(1), 34–42. https://doi.org/10.1016/j.jclinepi.2006.03.012

AUC, area under the curve; OR, odds ratio; CFA, confirmatory factor analysis; CFI, comparative fit index; COSMIN, consensus-based standard for the selection of health measurement instruments; CTT, classical test theory; DIF, differential item functioning; ICC, intraclass correlation coefficient; IRT, item response theory; LoA, limits of agreement; MIC, minimal important change; RMSEA, root mean square error of approximation; SDC, smallest detectable change; SRMR, standardised root mean residuals; TLI, Tucker-Lewis Index; GRADE, Grading of Recommendations, Assessment, Development, and Evaluations.

+, sufficient; −, insufficient; ?, indeterminate; ‡, good; ±, adequate; x, poor;

a , To rate the quality of the summary score, the factor structures should be equal across studies;

b , Unidimensionality refers to a factor analysis per subscale, while structural validity refers to a factor analysis of a (multi-dimensional) patient-reported outcome measure;

c , As defined by grading the evidence according to the GRADE approach;

d , This evidence may come from different studies;

e , The results of all studies should be taken together, and it should then be decided if 75% of the results are in accordance with the hypotheses;

f , Schreiber et al. (2006).

The COSMIN guidelines provide a consensus framework to compare psychometric properties of measures in a systematic manner. This article intends to apply the principles of this systematic process to the outcomes of psychometric analyses of multiple measures by using recent SAN samples. The context – assessment of resilience in the SAN – is used as an illustrative case study; the same principles could equally apply to other psychological measurements or social contexts as well.

### Aim

The first aim of the article was to explore psychometric characteristics of resilience-related measures among SAN populations, in order to consider evidence of local validity. Three specific objectives were pursued. Firstly, the study investigated structural validity indices, including dimensionality, measurement invariance, internal consistency and socio-demographic effects. Secondly, it investigated construct validity indices, by exploring associations with scales of common mental disorders (CMD) and perceived stress overload, as well as correlations between the resilience scales themselves. Thirdly, it investigated individual scale contributions to predicting (1) undesirable mental health outcomes and (2) emotional adaptation and self-rated performance during naval deployments.

The second aim was to demonstrate the application of systematic comparisons using COSMIN guidelines. To achieve this, it drew on both published and unpublished data from seven local SAN samples, across eight psychometric scales associated with resilience (and included the evidence generated from the first aim of this section). The samples and measures, as well as the relevant statistical analytical techniques are described in the ‘Methods’ section.

## Methods

### Process

Health research with the SAN is mainly carried out through the Institute for Maritime Medicine (IMM), which maintains comprehensive records of, among others, mental health data. This study drew on peer-reviewed published articles that dealt with resilience measures used in the SAN, and unpublished reports and datasets from the archives of IMM. To ensure reasonable recency, only data acquired within the past 5 years were included. The following eight scales were included: Brief Resilient Coping Scale (BRCS), Brief Sailor Resiliency Scale (BSRS), Connor–Davidson Resilience Scale (CD-RISC) 10- and 2-item versions, DRS-15, Mental Toughness Index (MTI-8) and Mental Toughness Questionnaire (MTQ) 18- and 6-item versions.

### Participants

Sample characteristics (e.g., size, age and gender composition) are reported in Table 2. Samples 1–4 represent unpublished archival data, while data from Samples 5–7 were previously published. All participants had at least a Grade 12 education. The samples were set up using a cross-sectional survey design.

**TABLE 2:** Socio-demographic and validity data across seven samples and eight measures.

Sections	BRCS (sample 3)	BSRS^a^ (sample 7)	BSRS^c^ (sample 5)	CD-RISC-10 (sample 3)	CD-RISC-2 (sample 6)	DRS-15^b^ (sample 5)	DRS-15^c^ (sample 2)	MTI-8 (sample 2)	MTI-8 (sample 3)	MTQ-18 (sample 1)	MTQ-18 (sample 2)	MTQ-18 (sample 4)	MTQ-18^c^ (sample 5)	MTQ-18^b^ (sample 6)	MTQ-6 (sample 3)
**Sample**															
*N*	729	1312	160	730	1880	1008	168	376	730	1123	433	321	168	893	729
Female (%)	32.6	21.4	22.9	32.6	25.9	29.9	22.9	26.1	32.6	28.9	26.8	15.8	22.9	32.4	32.6
Mean age	35.1	31.1	31.3	35.1	34.9	31.3	31.3	36.1	35.1	33	34.2	31.7	31.3	34.1	35.1
s.d.	8.5	7.6	6.4	8.5	8.7	8.4	6.4	10.1	8.5	9	10.6	6.2	6.4	9.2	8.5
Age range	20–62	20–62	21–64	20–62	20–64	20–59	21–64	20–64	20–62	20–59	20–64	21–59	21–64	19–59	20–62
Description	General navy	Deployed sailors	Deployed sailors	General navy	General navy	General navy	Deployed sailors	General navy	General navy	General navy	General navy	Deployed sailors	Deployed sailors	General navy	General navy
**Scale/measure**															
Mean	16.5	38.3	39.1	32.8	6.7	35.2	34.5	50.3	49.0	67.4	69.5	71.0	69.0	68.2	24.9
s.d.	2.2	6.4	6.2	5.3	1.3	-	5.5	5.8	6.1	10.1	8.6	7.8	8.2	-	3.1
Score range	8–20	-	26–54	13–40	0–8	15–45	18–45	13–56	12–56	29–90	36–90	45–90	45–90	29–90	6–30
**Structural validity**															
**Dimensionality**															
χ^2^/*df*	4.4±	3.3±	?	4.8±	?	?	?	5.1x	5.5x	4.8±	2.7‡	?	?	?	5.3x
RMSEA	0 068–,±	0.042+,‡	?	0 072–,±	?	?	?	0 08–,x	0 079–,±	0.058+,‡	0 062–,±	?	?	?	0 077–,±
CFI	0.99+,‡	0.998+,‡	?	0.957+,‡	?	?	?	0.947–,±	0.974+,‡	0.932–,±	0.871–,x	?	?	?	0.976+,‡
**Internal consistency**															
Cronbach α	0.746+,x	0.874+,‡	0.857+,‡	0.891+,‡	0.728+,x	0.714+,x	0.738+,x	0.887+,‡	0.897+,‡	0.870+,‡	0.825+,±	?	0.774+,x	0.884+,‡	0.847–,±
McDonald’s ω	0.746+,x	-	-	0.892+,‡	-	-	-	0.888+,‡	0.897+,‡	0.862+,‡	0.820+,±	-	-	-	0.847–,±
Inter-item *r*	0.40 – 0.53	-	-	0.30 – 0.64	0.57	-	-	0.28–0.69	0.31 – 0.73	0.01 – 0.58	0.01 – 0.45	-	-	-	0.43 – 0.62
Corrected item-total *r*	0.52–0.58	-	-	0.48 – 0.73	-	-	-	0.52–0.78	0.44 – 0.79	0.22 – 0.63	0.30 – 0.57	-	-	-	0.58 – 0.68
**Reliability**	?	?	?	?	?	?	?	?	?	?	?	?	?	?	?
**Measurement error**	?	?	?	?	?	?	?	?	?	?	?	?	?	?	?
**Demographic effects**															
Age (*r*)	0.088+	0.190*,–	?	0.154*,–	-	?	?	0.094–	0.183*,–	0.174*,–	0.174*,–	0.195*,–	?	?	0.140*,–
Gender	M > W	M > W	?	M = W	M = W	?	?	M = W	M > W	M = W	M = W	M > W	?	?	M > W
Difference (pt)	1.5	1.5	-	-	-	-	-	-	1.5	-	-	3	-	-	1
Language	E = N-E+	?	?	E = N-E+	E = N-E+	?	?	E = N-E+	E = N-E+	E = N-E+	E = N-E+	?	?	?	E = N-E+
**Measurement invariance**															
**Gender**															
Configural	+,‡	?	?	+,‡	?	?	?	+,‡	+,‡	+,‡	+,‡	?	?	?	+,‡
Metric	+,‡	?	?	+,‡	?	?	?	–,x	+,‡	–,x	+,‡	?	?	?	+,‡
**Language**															
Configural	+,‡	?	?	+,‡	?	?	?	+,‡	+,‡	+,‡	+,‡	?	?	?	+,‡
Metric	+,‡	?	?	+,‡	?	?	?	+,‡	+,‡	+,‡	+,‡	?	?	?	+,‡
**Construct validity**															
**Correlations**	-	-	-	-	-	-	-	-	-	-	-	-	-	-	-
CD-RISC-10 (*r*)	0.540*,+,‡	-	-	-	-	-	-	–	0.633*,+,‡	-	–	-	-	-	0.629*,+,‡
CD-RISC-2 (*r*)	-	-	-	-	-	-	-	0.380*,+,±	-	-	0.435*,+,±	-	-	-	-
MTQ–18 (*r*)	-	-	-	-	0.435*,+,±	-	-	0.507*,+,‡	-	-	-	-	-	-	-
MTQ–6 (*r*)	0.561*,+,‡	-	-	0.629*+,‡	-	-	-	-	0.532*,+,‡	-	-	-	-	-	-
MTI-8 (*r*)	0.518*,+,‡	-	-	0.633*+,‡	0.380*,+,±	-	-	-	-	-	0.507*,+,‡	-	-	-	0.532*,+,±
BRCS (*r*)	-	-	-	0.540*+,‡	-	-	-	-	0.518*,+,‡	-	-	-	-	-	0.561*,+,±
**Correlations**	-	-	-		-	-	-	-	-	-	-	-	-	-	-
PHQ-9 (*r*)	−0.244*,+,x	-	-	−0.387*,+,±	−0.326*,+,±	-	-	−0.386*,+,±	−0.355*,+,±	-	−0.538*,+,‡	-	-	-	−0.366*,+,±
GAD–7 (*r*)	−0.235*,+,x	-	-	−0.411*,+,‡	−0.321*,+,±	-	-	−0.383*,+,±	−0.378*,+,±	-	−0.510*,+,‡	-	-	-	−0.360*,+,±
Stress Overload (*r*)	−0.285*,+, ±	-	-	−0.355*,+,±	−0.388*,+,±	-	-	−0.428*,+,‡	−0.322*,+,±	-	−0.568*,+,‡	-	-	-	−0.360*,+,±
BRUMS (*r*)	-	−0.48*,+,‡	−0.391*,+,±	-	-	-	−0.256*,+,±	-	-	-	-	-	−0.273*,+,±	-	-
**Differentiate (Cohen’s *d*)**		-	-	-	-	-	-	-	-	-	-	-	-	-	-
MDD (*d*)	0.7*,–, x	-	-	1.7*,+,‡	1.2*,+,‡	-	-	1.4*,+,‡	1.3*,+,‡	-	1.5*,+,‡	-	-	-	1.3*,+,‡
GAD (*d*)	0.8*,–, x	-	-	1.6*,+,‡	1.6*,+,‡	-	-	2.2*,+,‡	1.4*,+,‡	-	2.0*,+,‡	-	-	-	1.4*,+,‡
**Responsivene (AUC)**		-	-		-	-	-	-	-	-	-	-	-	-	-
MDD	0.676–,x	-	-	0.863+,‡	0.722+,x	-	-	0.820+,±	0.816+,±	-	0.829+,±	-	-	-	0.804+,±
GAD	0.699–,x	-	-	0.866+,‡	0.829+,±	-	-	0.931+,‡	0.817+,±	-	0.948+,‡	-	-	-	0.826+,±
**Predictive validity**															
Odds ratios	-	-	-	-	-	-	-	-	-	-	-	-	-	-	
MDD	1.36±	-	-	1.31±	1.90‡	-	-	1.14 x	1.13 x	-	1.17 x	-	-	-	1.37±
GAD	1.40±	-	-	1.27±	2.11‡	-	-	1.16 x	1.13 x	-	1.23 ±	-	-	-	1.37±
**Performance during deployment (*β*-values)**															
Quality of work output (*β*)	-	-	-	-	-	-	-	-	-	-	-	-	< 0.10x	-	-
Quality of social interactions (*β*)	-	-	-	-	-	-	-	-	-	-	-	-	< 0.10x	-	-
Quality of emotion regulation (*β*)	-	-	-	-	-	-	-	-	-	-	-	-	< 0.20x	-	-
BRUMS (*β*)	-	-	0.41‡	-	-	-	-	< 0.20x	-	-	-	-	0.27±	0.43‡	-

*Source*: Superscript numbers refer to reference list:

a Van Wijk, C.H., & Martin, J.H. (2019). A Brief Sailor Resiliency Scale for the South African Navy. *African Journal of Psychological Assessment, 1*(3), a12. https://doi.org/10.4102/ajopa.v1i0.12;

b Arendse, D., Bester, P., & Van Wijk, C. (2020). Exploring psychological resilience in the South African Navy. In N.M. Dodd, P.C. Bester, & J. Van Der Merwe (Eds.), *Contemporary issues in South African military psychology* (pp. 137–160). African Sun Media.;

c Van Wijk, C.H. (2023). Dispositional resilience predicts psychological adaptation of seafarers during and after maritime operations. *International Maritime Health, 74*(1), 45-53. https://doi.org/10.5603/IMH.2023.0005

s.d., standard deviation; BRCS, brief resilient coping scale; BRUMS, brunel mood scale; BSRS, brief sailor resiliency scale; CD-RISC, connor-davidson resilience scale; CFI, comparative fit index; DRS, dispositional resilience scale; GAD, general anxiety disorder; MDD, major depressive disorder; MTI, mental toughness index; MTQ, mental toughness questionnaire; PHQ, patient health questionnaire for depression; RMSEA, root mean square error of approximation; M, men; W, women; E, English first language speakers; N-E, non-English first language speakers

*d*, Cohen’s *d*; *r*, coefficient.

* , *p* < 0.01.

+, sufficient; −, insufficient; ?, indeterminate; ‡, good; ±, adequate; x, poor.

#### Sample 1

Sample 1 was used to investigate the structural validity of the MTQ-18 by examining its psychometric characteristics in a general SAN sample of individuals from various occupational backgrounds and levels of experience, who were representative of the SAN. English as a first language was spoken by 25% of the sample. The detailed distribution of languages is presented in Appendix 1, Table 1-A1.

#### Sample 2

Sample 2, another general navy sample, completed the CD-RISC-2, MTI-8 and MTQ-18, and a subsample also completed other measures of mental health and general adjustment. The data were used to investigate the structural validity of the scales, as well as construct validity indices by exploring their association with measures of CMD and experience of stress overload, and finally exploring the utility of the scales to predict the presence of CMD. The sample was representative of the range of occupational fields and levels of experience in the SAN. English as a first language was spoken by 21% of the sample. Detailed distribution of language and occupational fields is presented in Appendix 1, Table 1-A1.

#### Sample 3

Sample 3, a general navy sample similar to Sample 2, was used in the same way to investigate the structural and construct validity of the CD-RISC-10, MTI-8 and MTQ-6. English as a first language was spoken by 19% of the sample. Distribution across language, qualification and occupational fields closely resembled that of Sample 2.

#### Sample 4

Successful emotional adaptation during shipboard deployments is critical for the wellbeing of individual sailors and the success of the mission, and Sample 4 was used to investigate the MTQ-18’s ability to predict performance during deployments, by exploring its association with self-rated performance and emotional regulation at the end of a 3-month operational deployment.

The sample comprised 321 volunteers who consented to complete the scales and questionnaires immediately prior to, and at the completion of a ship-based operational patrol of 3 months. Of the total group, 46.6% worked in combat-specific occupational fields, 31.1% in technical and engineering fields and 22.3% in support fields. All were experienced sailors.

#### Sample 5

South African Navy sailors who had been engaged in operational patrols completed the BSRS, DRS-15 and MTQ-18 prior to an operational cycle, and also provided measures of emotional regulation over the subsequent 12-month cycle. Further information can be found in Van Wijk (2023).

#### Sample 6

A general SAN sample completed the DRS-15 and MTQ-18, and the data were subjected to statistical analysis to explore their psychometric properties. Further information can be found in Arendse et al. (2020).

#### Sample 7

A sample of active-duty SAN sailors completed the BSRS for a validation study and provided socio-demographic information as well as measures of emotional regulation. Further information can be found in Van Wijk and Martin (2019).

### Measures

The eight resilience-related measures are briefly described first, and thereafter the other measures of mental health, stress overload, and emotional regulation that were used to evaluate construct and predictive validity. All eight measures were scored on Likert scales, with higher scores reflecting greater resilience, and all were administered in their standard, paper-based, English formats.

#### Brief Resilient Coping Scale

The four-item BRCS was designed to capture an individual’s ability to cope with stress in adaptive ways (Sinclair & Wallston, 2004). Evidences of acceptable reliability and validity have previously been reported, including Cronbach’s α = 0.68 (Sinclair & Wallston, 2004). It was completed by Sample 3.

#### Brief Sailor Resiliency Scale

The 12-item BSRS (Van Wijk & Martin, 2019) is a self-report measure of readiness for military duty, captured across mental, physical, social and spiritual domains. Good internal consistency and support for a four-factor structure have been reported, together with support for construct validity, for both SAN sailors (Van Wijk & Martin, 2019) and SA Army soldiers (Schoeman & Cassimjee, 2022). It was completed by Samples 5 and 7.

#### Connor–Davidson Resilience Scale – 10

The 10-item CD-RISC (Campbell-Sills & Stein, 2007) is a shortened version of the original 25-item CD-RISC (Connor & Davidson, 2003), with scores ranging from 0 to 40. Adequate reliability and validity have been reported (Campbell-Sills & Stein, 2007). A previous SA study (Pretorius & Padmanabhanunni, 2022) reported good internal consistency (Cronbach’s α = 0.95) and support for a unidimensional model. The SA student mean was closely aligned with the original validation study mean, and scores were negatively correlated to measures of depression and anxiety. It was completed by Sample 3.

#### Connor–Davidson Resilience Scale – 2

The two-item CD-RISC (Vaishnavi et al., 2007) is another shortened version of the 25-item CD-RISC (Connor & Davidson, 2003), and it uses two items from the original scale that were deemed to etymologically capture the essence of resilience (Vaishnavi et al., 2007). The CD-RISC – 2 scores are reportedly not affected by age, gender or race. They are also significantly correlated with measures of hardiness and perceived stress. Furthermore, these scores can differentiate between psychiatric outpatients and the general population (Vaishnavi et al., 2007). Adequate reliability and validity have been reported (Vaishnavi et al., 2007). It was completed by Sample 2.

#### Dispositional Resilience Scale – 15

This is one of the most used scales in military contexts across nations and languages (Bartone, 1999, 2000; Bartone & Homish, 2020; Britt et al., 2001; Escolas et al., 2013; Maddi & Harvey, 2006). However, previous applications in the South African National Defence Force (SANDF) found limited support for further use in its current form (Arendse et al., 2020). Scores for the 15-item scale range from 0 to 45, and six items are reverse scored. Good criterion-related validity across the United States (US) samples has been reported with Cronbach’s α > 0.8 for the full scale (Bartone, 1996, 1999), and support for the three hardiness dimensions observed (Hystad et al., 2010). It was completed by Samples 5 and 6.

#### Mental Toughness Index – 8

The MTI-8 reflects a unidimensional understanding of mental toughness, which plays an important role in performance, goal progress and thriving despite stress; and the scale has enduring properties across situations and time (Gucciardi et al., 2015). Scores for the eight-item scale range from 8 to 56. High model fit indices and reliabilities supporting a unidimensional model have been reported with Cronbach’s α and MacDonald’s ω > 0.8 (Gucciardi et al., 2015, 2021). Cross-cultural invariance of the MTI-8 has previously been established (Moreira et al., 2021; Stamatis et al., 2021). It was completed by Samples 2 and 3.

#### Mental Toughness Questionnaire – 18

The 18-item scale is a shortened version of the original MTQ-48 that taps a multi-dimensional understanding of mental toughness (Clough et al., 2002), with scores ranging from 18 to 90. Nine items are reverse-scored. Original reports of the MTQ-18 suggested a single-factor structure (Clough et al., 2002; Gerber et al., 2013, 2015), although one study extracted four factors aligned to the four dimensions of the MTQ-48 (Godlewski & Kline, 2012). Other studies did not manage to find a clear factor structure (Arendse et al., 2020; Dagnall et al., 2019). Cronbach’s α > 0.70 was previously reported, as was the lack of significant differences between gender groups (Clough et al., 2002; Gerber et al., 2013, 2015). Gender invariance at the configural, metric and scalar levels has also been demonstrated (Dagnall et al., 2019). It was completed by Samples 1, 2, 4, 5 and 6.

#### Mental Toughness Questionnaire – 6

The MTQ-6 is another shortened version of the original MTQ-48 (Clough et al., 2002) and consists of six items selected because of the best core-dimension definition (Kawabata et al., 2021). Scores range from 6 to 30. The six items exclude the reverse-scored items of the MTQ-18/48 to avoid potential wording effects (Wang et al., 2014). The MTQ-6 has demonstrated an excellent unidimensional fit, adequate internal consistency (e.g., Cronbach’s α and McDonald’s ω = 0.72) and measurement invariance for gender at a configural and metric level. The MTQ-6 has been significantly and negatively correlated to a measure of perceived stress (Kawabata et al., 2021). It was completed by Sample 3.

#### Indicators of common mental disorders

For Samples 2 and 3, CMD were identified as follows. The Patient Health Questionnaire for depression (PHQ-9; Gilbody et al., 2007) was used to screen for depression, with scores ≥ 10 used for identifying cases (Sample 2: *N* = 1880, Cronbach’s α = 0.83, McDonald’s ω = 0.84 and Sample 3: *N* = 730, Cronbach’s α = 0.84, McDonald’s ω = 0.85). The Generalised Anxiety Disorder scale (GAD-7; Löwe et al., 2008) was used to screen for generalised anxiety disorder, with scores ≥ 10 identifying cases (Sample 2: *N* = 1880; Cronbach’s α = 0.87, McDonald’s ω = 0.88 and Sample 3: *N* = 730, Cronbach’s α = 0.88, McDonald’s ω = 0.89).

#### Stress overload

A subgroup of Sample 2 (*N* = 430) also completed the 10-item Stress Overload Scale – Short Form (Amirkhan, 2018; Cronbach’s α and McDonald’s ω = 0.93 for this sample). Evidence of validity in the local SA context has previously been demonstrated (Van Wijk, 2021). Sample 3 completed the single-item Visual Analogue Scale for stress overload, which is scored on a 10-point visual analogue scale. For both scales, higher scores indicate respondents’ increased perception that the demands of their lives are overwhelming their available resources.

#### Brunel Mood Scale

The BRUMS (Terry et al., 2003) was used to measure emotional regulation. The total mood distress score – where higher scores represent poorer emotional regulation – was used (scores range from –16 to 80). The BRUMS has previously been used as a marker of mental health (Brandt et al., 2016) and to predict post-traumatic stress symptoms after maritime interdiction operations (Van Wijk et al., 2013). Good concurrent and criterion validity has been reported (Terry et al., 2003). The 20-item BRUMS (which excluded the Confusion subscale) was administered in English and completed by Samples 4 (Cronbach’s α = 0.80), 5 and 7.

#### Self-report assessment of performance

At the end of the mission, participants in Sample 4 were invited to rate their performance using a three-item scale, which referred to the quality of work output, interpersonal interactions and emotional state, over the past 6 weeks.

### Data analysis

For published articles (Samples 5–7; Arendse et al., 2020; Van Wijk, 2023; Van Wijk & Martin, 2019), the reports of applicable statistical results were directly transferred to Table 2. Samples 1–3 were subjected to the analysis in this section (where applicable). All statistical analyses were conducted by means of Statistical Package for Social Sciences (IBM SPSS for Windows, version 27) and analysis of moment structures (AMOS).

Effects of socio-demographic variables were explored using Pearson’s correlation coefficients for age, and *t*-tests for independent samples for gender and language. For this analysis, language was coded into two groups, namely English first language and not-English first language. Internal consistencies were examined with Cronbach’s α, MacDonald’s ω, inter-item correlations and corrected item-total correlations.

Given the contradictory reports on the factor structure of the MTQ-18, the data of Sample 1 were first subjected to an exploratory factor analysis (EFA), using the maximum likelihood method. After Sample 1 established a two-factor model for the MTQ-18, confirmatory factor analyses (CFA) were conducted to test models with a unidimensional and possibly multi-factorial structure.

Confirmatory factor analyses are used to test whether the data fit a hypothesised measurement model (Marker, 2002). In this study, the Maximum Likelihood estimator was used to explore model fit. For a CFA, the global fit χ^2^ would ideally be small and not significant; but as this is rarely achieved in large samples, the root mean square error of approximation (RMSEA) and comparative fit index (CFI) were also considered. Bartlett’s test of sphericity and the Kaiser–Meyer–Olkin test were performed to assess whether the data were suitable for factor analysis. The CD-RISC-10, MTQ-6 and MTI-8 previously demonstrated unidimensional structures (Gucciardi et al., 2015; Kawabata et al., 2021; Pretorius & Padmanabhanunni, 2022), and CFA were used to test a unidimensional model for each scale (and also for the BRCS).

Measurement invariance refers to the generalisability element of construct validity (Putnick & Bornstein, 2016), and it is assessed when scores need to be compared across groups (e.g., gender and language). Scales need to be invariant with respect to the way in which the latent constructs are formed (configural invariance), and the indicators or items should load similarly on latent factors across the groups (metric invariance). The requirement for invariance is that the difference in global χ^2^ between hierarchical models is not significant. Measurement invariance was evaluated for gender (men and women) and language (English first language speakers and not-English first language speakers).

Construct validity was explored by, firstly, examining associations between the resilience-related scales among themselves, and secondly with scales of CMD (PHQ-9, which was also coded for the presence of Major Depressive Disorder [MDD] and GAD-7 also coded for the presence of Generalised Anxiety Disorder) and perceived stress overload. This was carried out using Pearson’s correlations.

Associations between resilience-related scales and two markers of poor mental health (i.e., the presence of MDD and GAD) were examined by conducting *t*-tests for independent samples. Positive findings of associations were explored further to determine the predictive utility of each scale to mental health conditions: a series of binomial logistic regressions were conducted, together with receiver operating/operator characteristics (ROC) curve analyses.

For Sample 4, additional Pearson’s correlation coefficients were calculated, and linear regression analysis (with MTQ-18 as a regressor) was used to predict both performance across the three self-report performance indicators and mood state scale.

### Application of consensus-based standard for the selection of health measurement instruments guidelines

The COSMIN parameter guidelines as shown in Table 1 (Prinsen et al., 2018) were applied to evaluate each piece of evidence, using the codes for sufficient (+), insufficient (–) or indeterminant (?), based upon the strength of the reported measurement property. However, after this evaluation, there was in some cases little to differentiate between the scales, and more nuanced criteria (also described in Table 1) were then applied to assist decision-making when choosing an instrument for a particular practical application. It used the codes good (‡), adequate (±) and poor (x).

### Ethical considerations

This study used retrospective data, anonymised prior to inclusion in the final analyses. The project has been approved by the Health Research Ethics Committee of Stellenbosch University (reference no.: N20/07/078).

## Results

**FIGURE 1-A1:**
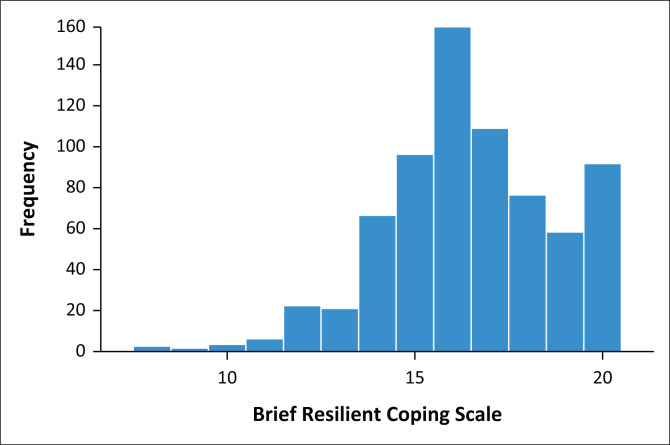
Sample 3, Brief Resilience Coping Scale mean score distribution.

**FIGURE 8-A1:**
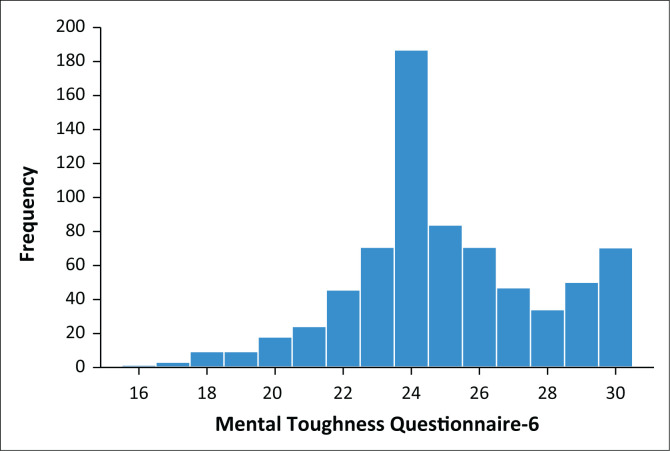
Sample 3, Mental Toughness Questionnaire-6 mean score distribution.

Statistical results for the eight scales across seven samples are summarised in Table 2, with additional statistical results presented in this section. The mean score distributions for the eight scales are graphically represented in Appendix 1, Figures 1–A1 to Figure 8–A1. The correlation matrix for each scale was adequate for factor analysis (Appendix 1, Table 2–A1). For scales where analyses were available, mean scores differentiated between individuals with positive responses on the mental health indicators and those without (Table 3).

**TABLE 3:** *T*-test for independent samples for resilience measures and indicators of common mental disorders.

Indicator	No			Yes			*t*	*p*	Cohen’s *d*
	*n*	*M*	s.d.	*m*	*M*	s.d.			
**Sample 2**									
**CD-RISC-2**									
MDD	1793	6.76	1.2	87	5.44	1.8	9.843	< 0.001	1.18
GAD	1853	6.72	1.2	27	4.89	1.5	6.425	< 0.001	1.59
**MTI-8**									
MDD	360	50.63	5.5	16	42.75	7.5	5.508	< 0.001	1.40
GAD	370	50.50	5.6	6	38.00	7.0	4.342	0.007	2.20
**MTQ-18**									
MDD	413	70.88	8.0	20	57.90	10.8	5.000	< 0.001	1.50
GAD	426	69.80	8.3	7	52.86	6.6	6.685	< 0.001	2.00
**Sample 3**									
**BRCS**									
MDD	696	16.55	2.2	33	14.94	2.5	3.573	< 0.001	0.70
GAD	707	16.53	2.2	22	14.73	2.5	3.335	0.003	0.80
**CD-RISC-10**									
MDD	697	33.18	4.9	33	24.67	5.8	8.286	< 0.001	1.70
GAD	708	33.05	5.1	22	24.77	5.6	6.863	< 0.001	1.60
**MTI-8**									
MDD	697	49.38	5.8	33	41.70	7.0	7.378	< 0.001	1.30
GAD	708	49.29	5.8	22	40.86	7.6	6.610	< 0.001	1.40
**MTQ-6**									
MDD	696	25.07	3.0	33	21.30	3.6	5.892	< 0.001	1.30
GAD	707	25.03	3.0	22	20.82	3.7	5.269	< 0.001	1.40

BRCS, brief resilience coping scale; CD-RISC, connor-davidson resilience scale; GAD, generalised anxiety disorder; MDD, major depressive disorder; MTI, mental toughness index; MTQ, mental toughness questionnaire.

### Brief Resilient Coping Scale (Sample 3)

There was a significant difference in the BRCS mean scores of women and men (Table 4), with men scoring on average 1.5 points higher. There was no significant difference in the mean scores of English first language and non-English first language speakers (Table 4).

**TABLE 4:** *T*-test for independent samples for gender and language across measures and samples.

Measure	Sample	Gender			Language		
		*t*	*p*	*d*	*t*	*p*	*d*
BRCS	3	3.061	< 0.001	0.30	0.003	0.499	< 0.1
BSRS	5	4.160	< 0.001	0.28	-	-	-
CD-RISC-10	3	2.410	< 0.050	0.20	0.283	0.777	< 0.1
CD-RISC-2	2	2.455	< 0.050	0.10	2.810	< 0.01	0.2
MTI-8	2	1.474	0.143	0.20	1.079	0.282	0.1
MTI-8	3	3.065	< 0.050	0.30	0.000	1.000	-
MTQ-18	1	3.669	< 0.001	< 0.10	4.293	< 0.001	0.3
MTQ-18	2	1.870	0.063	0.02	1.154	0.251	0.1
MTQ-18	4	2.729	< 0.010	0.40	-	-	-
MTQ-6	3	5.007	< 0.001	0.40	0.403	0.344	< 0.1

BRCS, brief resilience coping scale; BSRS, brief sailor resiliency scale; CD-RISC, connor-davidson resilience scale; MTI, mental toughness index; MTQ, mental toughness questionnaire; *d*, Cohen’s *d*.

While the 1-factor model did not obtain a non-significant χ^2^ (χ^2^ = 8.765, *df* = 2, *p* < 0.05) during CFA, the RMSEA (0.068; 90% CI: 0.027–0.117) was adequately small and the CFI (0.990) supported an adequate fit. Standardised loadings ranged from 0.60 to 0.73. The BRCS unidimensional model showed acceptable configural and metric invariance for gender (Δχ^2^ = 0.668, Δ*df* = 13, *p* = 0.881) and language (Δχ^2^ = 7.238, Δ*df* = 3, *p* = 0.065).

The BRCS orrelated significantly with other scales measuring resilience, CMD and stress overload. The binomial logistic regressions for all the indicators were statistically significant (Table 5), but none showed meaningfully raised odds ratios. Neither did the ROC analysis report any clinically useful areas under the curve.

**TABLE 5:** Binomial regression for resilience measures and indicators of common mental disorders and other adjustment difficulties.

Indicator	Nagelkerke *R*^2^ (% variance explained)	*χ* ^2^	PAC	Wald	OR	95% CI	AUC
**Sample 2**							
**CD-RISC-2**							
MDD	12.4	74.506*	95.4	75.109*	1.90	1.64–2.19	0.722
GAD	15.4	40.851*	98.6	45.403*	2.11	1.70–2.63	0.829
**MTI-8**							
MDD	15.1	17.243*	95.7	16.235*	1.14	1.07–1.21	0.820
GAD	22.1	12.788*	98.4	13.679*	1.16	1.07–1.25	0.931
**MTQ-18**							
MDD	25.7	36.264*	95.4	29.415*	1.17	1.11–1.24	0.829
GAD	34.8	23.591*	98.4	16.887*	1.23	1.11–1.36	0.948
**Sample 3**							
**BRCS**							
MDD	7.0	15.875*	95.5	16.075*	1.36	1.17–1.58	0.676
GAD	7.6	13.301*	97.0	13.741*	1.40	1.17–1.67	0.699
**CD-RISC-10**							
MDD	29.9	70.485*	95.3	55.494*	1.31	1.22–1.40	0.863
GAD	24.5	43.617*	97.3	38.896*	1.27	1.18–1.37	0.866
**MTI-8**							
MDD	14.5	33.354*	94.8	32.773*	1.13	1.09–1.18	0.816
GAD	14.4	25.286*	96.4	27.593*	1.13	1.08–1.18	0.817
**MTQ-6**							
MDD	16.5	38.128*	95.2	34.106*	1.37	1.23–1.52	0.804
GAD	17.2	30.285*	97.0	28.457*	1.37	1.22–1.54	0.826

BRCS, brief resilience coping scale; CD-RISC, connor-davidson resilience scale; MTQ, mental toughness questionnaire; MTI, mental toughness index; PAC, percentage accuracy in classification; OR, odds ratio; 95% CI, 95% confidence interval; AUC, area under the curve.

* , *p* < 0.01.

### Brief Sailor Resiliency Scale (Samples 5 and 7)

In summary, Sample 7 provided evidence of acceptable model fit: χ^2^ = 159.59, *df* = 48, *p* < 0.001; RMSEA = 0.042 (95% CI: 0.035–0.049) and CFI = 0.998. Men scored on average 1.8 points higher than women (Table 4), and the BSRS correlated significantly with a measure of emotional regulation (Van Wijk & Martin, 2019). Sample 5 further provided evidence that the BSRS can predict emotional regulation during and at the end of shipboard deployments (Van Wijk, 2023).

### Connor–Davidson Resilience Scale-10 (Sample 3)

The CD-RISC-10 mean score (32.8) was about 1 standard deviation higher than both the SA student sample (*M* = 26.9, *t* = 30.250, *p* < 0.001, *d* = 1.1; Pretorius & Padmanabhanunni, 2022) and the original validation study (*M* = 27.2, *t* = 28.710, *p* < 0.001, *d* = 1.1; Campbell-Sills & Stein, 2007). There was a significant difference in the CD-RISC-10 mean scores of women and men (Table 4), with the actual differences in scores negligible. There was no significant difference in the mean scores of English first language and non-English first language speakers (Table 4).

A 1-factor model did not obtain a non-significant χ^2^ (χ^2^ = 168.093, *df* = 35, *p* < 0.001) during CFA, but the RMSEA (0.072; 90% CI: 0.061–0.083) was adequately small and the CFI (0.957) supported an adequate fit. Standardised loadings ranged from 0.50 to 0.78. The CD-RISC-10 unidimensional model showed acceptable configural and metric invariance for gender (Δχ^2^ = 13.261, Δ*df* = 9, *p* = 0.151) and language (Δχ^2^ = 15.3741, Δ*df* = 9, *p* = 0.081).

The CD-RISC-10 correlated significantly with other scales measuring resilience, CMD and stress overload. The binomial logistic regressions for all the indicators were statistically significant (Table 5), but none showed meaningfully raised odds ratios. Clinically useful (> 80%) areas under the curve were reported for MDD and GAD.

### Connor–Davidson Resilience Scale-2 (Sample 2)

There was a significant difference in the CD-RISC-2 mean scores of women and men, as well as in the scores of English first language and non-English first language speakers (Table 4). In both cases, the effect sizes were very small, and the actual mean score differences were negligible.

The CD-RISC-2 correlated significantly with other scales measuring resilience, CMD and stress overload. The binomial logistic regressions for all the indicators were statistically significant (Table 5), with an OR > 1.5, implying that lower resilience was associated with increased odds for undesirable mental health outcomes. A clinically useful area under the curve was reported for GAD.

### Dispositional Resilience Scale-15 (Samples 5 and 6)

In summary, Sample 6 reported problematic structural validity. While a 3-factor solution provided the best fit, it did not correspond to the three theoretical facets, and questionable internal consistency was further reported (Arendse et al., 2020). The DRS-15 failed to predict emotional regulation during or after shipboard deployments (Sample 5, Van Wijk, 2023).

### Mental Toughness Index-8 (Samples 2 and 3)

For Sample 2, there was no significant difference in the MTI-8 mean scores of women and men or English first language and non-English first language speakers (Table 4). For Sample 3, there was a significant difference in the MTI-8 mean scores of women and men, with men scoring on average 1.5 points higher, but again there were no significant differences between the mean scores of English first language and non-English first language speakers (Table 4).

Sample 2 data were subjected to CFA. Although the 1-factor model did not obtain a non-significant χ^2^ (χ^2^ = 102.103, *df* = 20, *p* < 0.001), the value was not excessively high and the CFI (0.947) did suggest an adequate fit. However, the RMSEA (0.080; 90% CI: 0.070–0.090) was only marginally supportive. Standardised loadings were relatively uniform, ranging from 0.56 to 0.83.

Sample 3 data were also subjected to CFA. While the 1-factor model did not obtain a non-significant χ^2^ (χ^2^ = 110.098, *df* = 20, *p* < 0.001), the RMSEA (0.079; 90% CI: 0.065–0.093) was adequately small and the CFI (0.974) supported an adequate fit. Standardised loadings ranged from 0.46 to 0.85.

In Sample 2, the unidimensional model showed acceptable configural invariance for gender but did not reach metric invariance (Δχ^2^ = 14.363, Δ*df* = 7, *p* = 0.045), while the model showed acceptable configural and metric invariance for language (Δχ^2^ = 6.113, Δ*df* = 7, *p* = 0.527). In Sample 3, the unidimensional model showed acceptable configural and metric invariance for gender (Δχ^2^ = 6.500, Δ*df* = 7, *p* = 0.483) and language (Δχ^2^ = 4.420, Δ*df* = 7, *p* = 0.730).

The MTI-8 in both Samples 2 and 3 correlated significantly with other scales measuring resilience, CMD and stress overload. The binomial logistic regressions for all the indicators were statistically significant (Table 5), but none showed meaningfully raised odds ratios. Clinically useful areas under the curve were reported for MDD and GAD.

### Mental Toughness Questionnaire-18 (Samples 1, 2, 4, 5 and 6)

For Sample 1, there was a significant difference in the MTQ-18 scores of women and men (Table 4), with men scoring higher. There was also a significant difference in the MTQ-18 scores of English first language and non-English first language speakers (Table 4), with English first language speakers scoring higher. In both cases, the actual differences in scores were negligible. Sample 2 found no significant differences in the mean scores of women and men or English first language and non-English first language speakers (Table 4). In contrast, Sample 4 found significant differences in the MTQ-18 full-scale scores of women and men (Table 4), with men scoring on average 3 points higher.

For Sample 1, the EFA, after varimax rotation, indicated a 2-factor solution as the best fit (Table 6), explaining 41.9% of the variance. No discernible item clustering according to theoretical concepts was observed. Rather, the items in the two factors were exactly aligned with the valence of the questions. Factor 1 consisted of items that were reverse-scored, while Factor 2 consisted of items that were not. Sample 6 reported a similar EFA with two factors accounting for 41% of the variance (Arendse et al., 2020).

**TABLE 6:** Mental Toughness Questionnaire-18 factor loadings.

Item	Factor 1	Factor 2
1	-	0.631
2	0.437	-
3	0.707	-
4	-	0.597
5	-	0.688
6	0.722	-
7	-	0.509
8	0.651	-
9	0.492	-
10	-	0.625
11	0.504	-
12	0.655	-
13	-	0.529
14	-	0.795
15	-	0.828
16	0.696	-
17	0.714	-
18	-	0.302
Cronbach’s α	0.857	0.852

Note: Extraction method, maximum likelihood; rotation, Varimax with Kaiser normalisation.

Confirmatory factor analyses were then conducted on Sample 1 data to test both 1- and 2-factor solutions. The 1-factor model obtained a significant χ^2^ (χ^2^ = 2874.092, *df* = 135, *p* < 0.001). The RMSEA (0.134; 90% CI: 0.130–0.139) and CFI (0.632) further indicated poor fit. Standardised loadings ranged from 0.25 to 0.66. The 2-factor model did not obtain a non-significant χ^2^ either (χ^2^ = 640.087, *df* = 134, *p* < 0.0001), but while not an absolute fit, the RMSEA (0.058; 90% CI: 0.054–0.063) was adequately small, and the CFI (0.932) also supported an adequate fit. Standardised loadings for factor 1 ranged from 0.43 to 0.75 and from 0.30 to 0.83 for factor 2. The covariance between the two factors was 0.43. The 2-factor model appeared to have the best fit to the data.

For Sample 2, the 2-factor model was subjected to CFA. It did not obtain a non-significant χ^2^ (χ^2^ = 354.691, *df* = 134, *p* < 0.001). The RMSEA (0.062; 90% CI: 0.054–0.070) was adequately small, but the CFI (0.871) did not support an adequate fit. Standardised loadings for factor 1 ranged from 0.38 to 0.71, and from 0.13 to 0.62 for factor 2. The covariance between the two factors was 0.66.

For Sample 1, the 2-factor model showed acceptable configural invariance for gender but did not achieve metric invariance (Δχ^2^ = 33.319, Δ*df* = 16, *p* = 0.007). The 2-factor model showed acceptable configural and metric invariance for language (Δχ^2^ = 19.611, Δ*df* = 16, *p* = 0.238). Similarly, for Sample 2, the 2-factor model showed acceptable configural invariance for gender but did not achieve metric invariance (Δχ^2^ = 31.109, Δ*df* = 16, *p* = 0.009), while the model showed acceptable configural and metric invariance for language (Δχ^2^ = 18.388, Δ*df* = 16, *p* = 0.302).

The MTQ-18 correlated significantly with other scales measuring resilience, CMD and stress overload. The binomial logistic regressions for all the indicators were statistically significant (Table 5), but none showed meaningfully raised odds ratios. Clinically useful areas under the curve were reported for MDD and GAD.

The correlations between MTQ-18 scores (Sample 4) and self-report performance and emotional regulation among a group of deployed sailors are presented in Table 7. Mental toughness correlated significantly to both self-rated performance and self-reported mood states, with modest effect sizes. However, during linear regression analysis, it predicted emotional regulation during deployment only, with a modest effect size (Table 2). The MTQ-18 was also able to predict emotional regulation during and after operational cycles (Sample 5, Van Wijk, 2023).

**TABLE 7:** Correlations between mental toughness and self-rated performance and mood states at the end of deployment.

Measure	*N*	Full scale	
		*r*	*p*
Quality of work output	151	0.285	< 0.001
Quality of interpersonal interactions	151	0.301	< 0.001
Quality of emotional state	151	0.350	< 0.001
Brunel mood scale	314	−0.406	< 0.001

### Mental Toughness Questionnaire-6 (Sample 3)

There was a significant difference in the MTQ-6 mean scores of women and men (Table 4), with men scoring on average 1 point higher. There was no significant difference in the mean scores of English first language and non-English first language speakers (Table 4).

The 1-factor model did not obtain a non-significant χ^2^ (χ^2^ = 48.126, *df* = 9, *p* < 0.001) during CFA, but the RMSEA (0.077; 90% CI: 0.057–0.099) was adequately small and the CFI (0.976) supported an adequate fit. Standardised loadings ranged from 0.62 to 0.76. The MTQ-6 unidimensional model showed acceptable configural and metric invariance for gender (Δχ^2^ = 8.965, Δ*df* = 5, *p* = 0.110) and language (Δχ^2^ = 7.492, Δ*df* = 5, *p* = 0.187).

The MTQ-6 correlated significantly with other scales measuring resilience, CMD and stress overload. The binomial logistic regressions for all the indicators were statistically significant (Table 5), but none showed meaningfully raised odds ratios. Clinically useful areas under the curve were reported for MDD and GAD.

### Consensus-based standard for the selection of health measurement instruments outcomes

The COSMIN outcome codes, as well as the nuanced codes to aid further decision-making are presented in Table 2. On the surface, there was little to differentiate between the measures, with a number of scales offering acceptable psychometric properties in the context. After considering the nuanced coding, four scales, namely the BSRS, CD-RISC-10, MTI-8 and MTQ-6 appeared marginally superior, while the BRCS and DRS-15 displayed questionable properties in this context. This assessment was based on the characteristics of internal consistency, dimensionality and ability to differentiate mental health states (Table 2).

## Discussion

### Psychometric characteristics of the identified resilience-related measures

As discussed, there was relatively little to differentiate between the scales’ psychometric characteristics. The scales correlated significantly with related scales in their respective samples, as well as with the mental health screeners, in the expected direction. Where tested, scales differentiated between sailors with CMD and those without. These findings provide support for the construct validity of the identified measures.

The BSRS and CD-RISC-10 showed acceptable structural validity, and the MTQ-6 and MTI-8 presented marginally acceptable results, while the MTQ-18 was more inconsistent in its evidence. The BRCS, CD-RISC-2 and DRS-15, in general, did not meet the more stringent criteria at this time. This may be partly because of missing statistical indicators across all the measures, and more work would be required to conclusively compare the eight scales.

The BSRS, CD-RISC and MTQ-6 offered some evidence of the ability to predict outcomes. The MTI-8 and MTQ-18 again showed inconsistent results, while the BRCS and DRS-15 did not meet the criteria of acceptability. However, much of the data were retrospective in nature, which limits the interpretation of any actual ‘predictive’ results. Prospective studies, using real-world challenging experiences (such as long-range deployments), would be important to further the understanding of the relationship between resilience and other psychological outcomes, and the eventual practical value of resilience measures in this context.

The CD-RISC-10 mean scores were significantly higher than those of SA students and original US validation samples and could arguably reflect a normative naval resilient sample. The higher resilience scores could be hypothesised to be because of participants meeting SANDF entry criteria, as well as the development of resilience through experience. Similar observations could potentially be possible for the other scales, where direct comparative norm-data were not available. Interestingly, for all measures represented in more than one sample, mean scores were similar across those samples, suggesting some stability of mean score values within the larger SAN population.

### Gender and context

There were some inconsistencies with regard to gender effects. In some cases, the mean score difference between women and men (irrespective of whether significant or not) was very small and would have little practical implication during interpretation. In other cases, the differences were large enough to affect interpretation. Further sampling might clarify this finding.

It was noteworthy that the most substantial gender difference was observed for mental toughness among the ship-on-patrol participants (Samples 4 and 7). This may speak to the role of context in the following way. While the SAN’s aggressive policies on gender mainstreaming are thought to have reduced the hyper-gendered nature of general navy business, deployed settings (ships or otherwise) are still highly gendered environments (Martin & Van Wijk, 2020; Richard & Molloy, 2020). It could be hypothesised that the (perceived) expectation of men to portray themselves in (hyper)masculine ways, and the (perceived) expectation of women to remain feminine (Martin & Van Wijk, 2020; Richard & Molloy, 2020) are reflected in their reported mental toughness. Thus, in a general SAN sample, there was little actual gender difference in mean scores, but on ships as a ‘gendered’ environment, substantial differences were still observed. At the nexus of gender and the military, context matters.

### Language

English was not the first language for the greater proportion of participants. Yet, configural and metric invariance for language has been observed across all scales (where available), and where actual differences in mean scores were found, they were very small and would have little practical implication during interpretation. A SANDF entry requirement is a matric certificate (≥ 12 years of formal schooling), and basic military and subsequent vocational training is conducted in English. Together, this seems to provide for sufficient English proficiency, and the scales appear appropriate for fair use in the SAN context, irrespective of sailors’ mother tongue.

The reverse scoring of items presents an interesting dilemma in multi-lingual psychometric assessment. Reverse-scored items serve a useful purpose in disrupting undesirable response sets, such as a systematic response bias through acquiescence. However, the benefits may be outweighed by the potential for methodologically induced bias. This would typically be visible in lower internal consistency, and lower inter-item correlations. Reverse-scored items commonly cluster into a separate factor, across a variety of populations and assessments. Factor analysis thus often supports a 2-factor solution against the unidimensionality of a measure, and while such factors can sometimes be interpreted substantively, their content typically co-varies with a reversed item format, raising the possibility that the loadings are at least partially methodologically based (Carlson et al., 2011; Dunbar et al., 2000; Marsh, 1986, 1996; Reise et al., 2007; Wang et al., 2014; Wong et al., 2003; Woods, 2006). This seems likely the case with the MTQ-18, where the apparent dimensionality is likely to be an artefact of the valence of the items, rather than reflecting two underlying constructs.

### Systematic comparisons through the application of consensus-based standards for the selection of health measurement instruments guidelines

The COSMIN criteria – as applied according to the guidelines in Table 1 – provided a framework to compare different measures purporting to tap resilience-related constructs. This was an important first step for a systematic comparison. The COSMIN criteria were developed for general application, across measures of different constructs and different populations. In the current comparison, many of the measures produced generally similar results. In such cases, therefore, these guidelines may be too general, and not nuanced enough to sufficiently differentiate between scales, particularly in the case of comparable samples (from the same population), or theoretically comparable measures. The current comprehensive systematic comparison further suffered from missing indices (e.g. reliability and measurement error), which may impede confident conclusions with regard to making practical recommendations.

In the context of African-focussed research, greater awareness of COSMIN (or another framework) guidelines would be necessary when designing local studies on psychometric measures. Further, a more nuanced grading of indices may be helpful when results are generally similar. In this study, the additional more stringent criteria (Table 1) were somewhat arbitrarily developed, for illustration purposes, and will thus benefit from a more formal articulation.

### Recommendation of scales and practical application

At this stime, two scales appear to have potential for practical use. The BSRS and CD-RISC-10 have well-developed theoretical underpinnings and displayed marginally superior measurement properties compared to the other scales. More work may be required, however, particularly regarding temporal stability and predictive utility, before applying them in practice with adequate confidence. Two further scales also seem to be worth further exploration in this context. The MTI-8 and MTQ-6 also have well-developed theoretical underpinnings, and while their statistical results were not as convincing, they are brief, use simple vocabulary and are invariant for language (in this context), which makes them attractive for use in settings where psychometric evaluation may become burdensome.

It is recognised that missing indices preclude confident final recommendations. Table 2 remains open to interpretation, and the data reported therein may allow policy makers in the naval health support context to make their own informed choices regarding which scales to use in practice. In doing so, the criteria set out for comparative analysis (including evidence for structural, construct and predictive validity) will need to be balanced by practical concerns (such as brevity, acceptance by respondents and so forth).

Such choices would be important, as the measurement of resilience in the SAN context has several applications, for both individual and organisational interventions (Van Wijk, 2023): Firstly, given the association with undesirable mental health and occupational outcomes, lower resilience may indicate risk and may warrant referral for early intervention. Identifying potentially vulnerable individuals to stream them towards support services could facilitate the development of greater resilience, possibly through context-appropriate skills training. Secondly, its association with psychological adaptation emphasises the value of enhancing resilience as a formal objective of military preparation. There are several ways to achieve this, such as through facilitating formal developmental experiences (military training courses; graded exposure to operational demands) and/or through mission-specific preparation programmes for sailors awaiting deployment. Thirdly, they could be used to measure the effectiveness of interventions (at the individual or military unit level) to enhance resilience.

### Limitations and future directions

The samples and analyses share two limitations. There was no information on their stability over time (e.g., no evidence of test-retest reliability), which would be important if the scales were to be used to measure change in resilience after intervention. There was further limited prospective predictive data available, which would be important to validate the use of such scales for predicting performance during deployments, or longer-term mental health. In this regard, prospective, longitudinal studies using actual deployments would enhance the understanding of the predictive utility of resilience measures for actual psychological performance both during and after maritime deployments. Samples 4 and 5 offered initial examples that can be built on.

The COSMIN guidelines might not be nuanced enough for scales reporting generally similar psychometric properties. Further work in articulating a more nuanced framework may be important to support systematic comparisons.

Lastly, expanding research across different but related populations – such as the SA Army or SA Air Force, SA Police Service, as well as emergency services or even private security companies – would aid in understanding the role of different settings in the relationship between resilience and psychological outcomes.

## Conclusion

This article illustrated the application of COSMIN guidelines for the systematic comparison of self-report resilience scales, using retrospective reports of SAN samples as a practical case study. It drew on both published and unpublished data from seven local SAN samples, across eight psychometric scales associated with resilience.

There was evidence for structural validity (ranging from good to marginally acceptable to problematic) across the eight scales, while positive evidence of good construct validity was found throughout. The association between resilience and emotional adaptation during and after maritime operations provided initial evidence of the ability of these scales to predict psychological adjustment in the context of naval deployments.

Although there was little evidence to differentiate definitively between the scales, the BSRS, CD-RISC-10, MTI-8 and MTQ-6 appear, for now, to have marginally better psychometric properties. This systematic comparison may allow policymakers to make informed choices with regard to the preferred use of scales.

## Figures and Tables

**FIGURE 2-A1:**
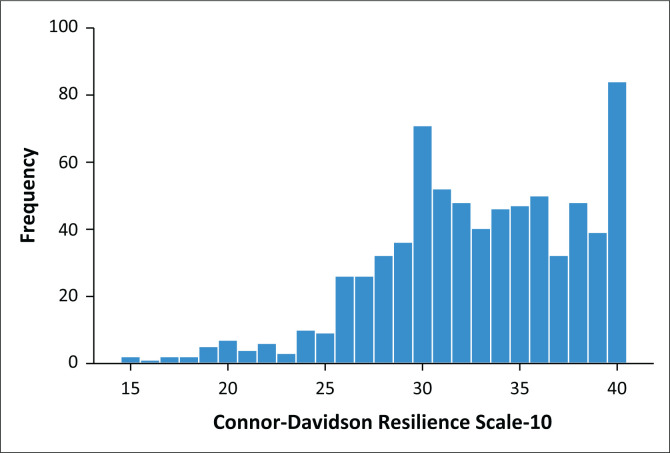
Sample 3, Connor-Davidson Resilience Scale-10 mean score distribution.

**FIGURE 3-A1:**
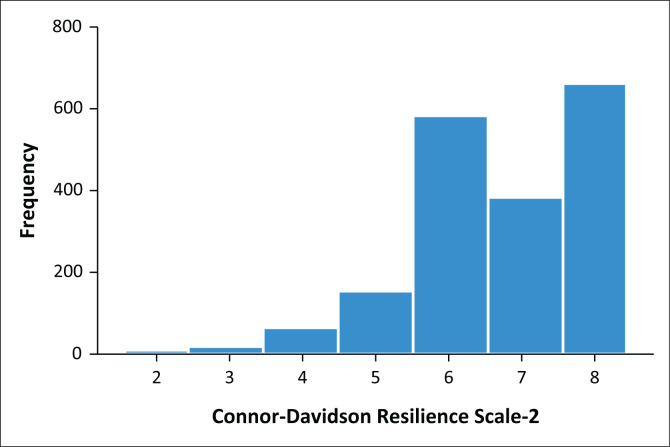
Sample 2. Connor-Davidson Resilience Scale-2 mean score distribution.

**FIGURE 4-A1:**
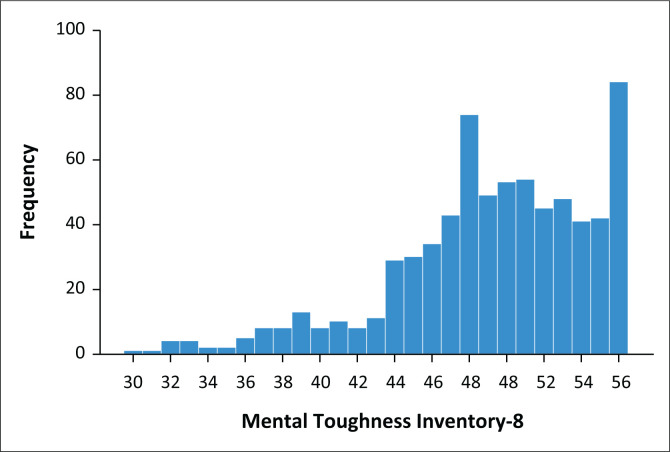
Sample 2, Mental Toughness Index-8 mean score distribution.

**FIGURE 5-A1:**
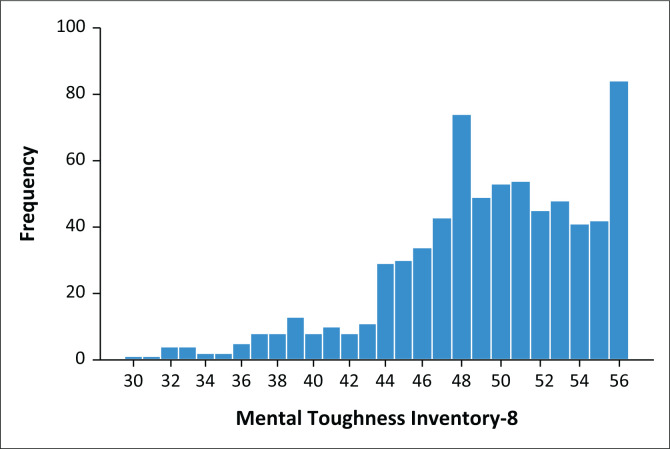
Sample 3, Mental Toughness Index-8 mean score distribution.

**FIGURE 6-A1:**
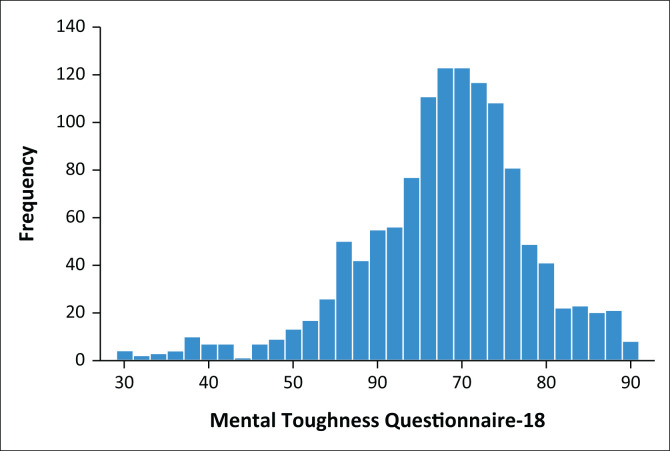
Sample 1, Mental Toughness Questionnaire-18 mean score distribution.

**FIGURE 7-A1:**
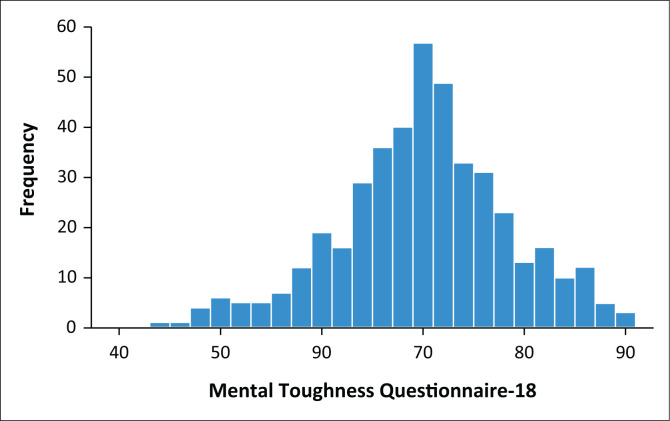
Sample 2, Mental Toughness Questionnaire-18 mean score distribution.

## APPENDIX 1

### Mean score distribution

**TABLE 1-A1:** Samples 1 and 2 distribution across home language and occupational field.

Variable	Sample 1		Sample 2	
	*n*	%	*n*	%
**Language**				
English	278	24.8	391	20.8
Afrikaans	208	18.5	320	17.0
isiZulu	169	15.0	223	11.9
IsiXhosa	123	11.0	217	11.5
Setswana	102	9.1	220	11.7
Sesotho	81	7.2	174	9.3
Sepedi	74	6.6	160	8.5
Tshivenda	28	2.5	69	3.7
Tsonga	20	1.8	40	2.1
SiSwati	19	1.7	39	2.1
Ndebele	16	1.4	27	1.4
Unknown	5	0.4	-	-
**Occupational sectors**				
Administrative/clerical	-	-	241	12.8
Technical/engineering	-	-	444	23.6
Naval combat specialist	-	-	495	26.3
Naval combat support	-	-	242	12.9
Marines	-	-	159	8.5
Other	-	-	299	15.9

**TABLE 2-A1:** Correlation matrix for factor analysis.

Sample	Scale	Bartlett’s test			Kaiser–Meyer–Olkin test
		χ^2^	*df*	*p*	
1	MTQ-18	7552.353	153	< 0.001	0.917
2	MTI-8	1573.381	28	< 0.001	0.907
	MTQ-18	1839.647	153	< 0.001	0.875
3	BRCS	650.097	6	< 0.001	0.761
	CD-RISC	3146.473	45	< 0.001	0.931
	MTI-8	3424.268	28	< 0.001	0.932
	MTQ-6	1614.543	15	< 0.001	0.876

Note: Adequacy of the correlation matrix for factor analysis indicated by a significant Bartlett’s test (*p* < 0.05) and a KMO index > 0.70.

BRCS, brief resilience coping scale; CD-RISC, connor-davidson resilience scale; MTI, mental toughness index; MTQ, mental toughness questionnaire; KMO, Kaiser-Meyer-Olkin.
